# Cerebro-Spinal Fever

**Published:** 1899-07-01

**Authors:** 


### CEREBRO-SPINAL FEVER.

The Cavendish Lecture delivered by Dr. William Osler on the Etiology and Diagnosis of Cerebro-Spinal Fever, interesting as it was as a clinical study, did not seem to advance matters very much beyond the point at which they before stood. What he showed pretty clearly was that epidemic cerebro-spinal fever is due to a specific micro-organism, the diplococcus intracellularis of Weichselbaum, and that some at least of the sporadic cases of cerebro-spinal meningitis, and probably the majority of the cases known as simple posterior basic meningitis of infants are due to the same micro-organism. There the matter stands. The next step would seem to be, working on the lines laid down by Dr. Osler, to differentiate according to their origin the various cases of sporadic meningitis, a not inconsiderable number of which appear to be due to infection by the pneumococcus, while some are, of course, due to pyogenic organisms.

